# Eclipsed Mitral Regurgitation: Paroxysmal Reversible Severe Mitral Regurgitation Presenting with Recurrent Pulmonary Edema: A Case Report

**DOI:** 10.7759/cureus.113718

**Published:** 2026-07-31

**Authors:** Naoki Sumi, Rikuto Nii, Yuhei Saitoh

**Affiliations:** 1 Department of Cardiovascular Surgery, Matsue Red Cross Hospital, Matsue, JPN

**Keywords:** acute pulmonary edema, case report, echocardiography, eclipsed mitral regurgitation, left ventricular ejection fraction, mitral valve replacement

## Abstract

Eclipsed mitral regurgitation (MR) is a rare, paroxysmal entity with preserved left ventricular ejection fraction, marked by abrupt, severe, but reversible MR that is demonstrable only during episodes. We report the case of an 82-year-old woman with systemic sclerosis and persistent atrial fibrillation who had four admissions for acute heart failure within six months. Transthoracic echocardiography captured massive MR with leaflet malcoaptation, severe pulmonary hypertension, a dagger-shaped MR jet, and a blood pressure drop; between episodes, MR was mild and leaflet coaptation was normal, and provocation testing was negative. Recurrent events during hospitalization prompted urgent surgery. Mitral valve replacement with concomitant tricuspid annuloplasty prevented recurrence, and the postoperative course was uneventful until death from tuberculosis-related sepsis. Operative inspection showed minimal structural change of the mitral valve, and histopathology demonstrated preserved leaflet architecture with mild fibrosis and myxomatous change, indicating no significant intrinsic valvular damage. These findings indicate that eclipsed MR is a unique pathophysiological entity.

## Introduction

Eclipsed mitral regurgitation (MR) is a rare, paroxysmal form of functional MR with preserved left ventricular ejection fraction (LVEF) [1]. It is characterized by transient, severe, and reversible regurgitation with a dramatic increase in severity during episodes and marked improvement or complete resolution between episodes [2]. Because events are transient, diagnosis requires echocardiographic documentation during an acute episode, and a negative provocation test does not exclude the diagnosis [3,4]. Given the heterogeneity and underrecognition of this entity, calls for standardized diagnostic criteria and treatment strategies have been made [5].

Although the underlying mechanism remains unclear, eclipsed MR differs from atrial functional MR - marked by chronic atrial enlargement, progressive annular dilation, and persistent MR - and from ventricular functional MR associated with ventricular remodeling [6,7]. While medical stabilization has been described in selected situations, recurrences are common and repeated pulmonary edema may necessitate surgery [8-10]. Here, we present a case of eclipsed MR associated with recurrent acute pulmonary edema in an elderly woman with systemic sclerosis, highlighting the diagnostic difficulty and practical therapeutic considerations.

## Case presentation

An 82-year-old woman with systemic sclerosis and a history of persistent atrial fibrillation, independent in activities of daily living, underwent four hospitalizations for acute heart failure over six months. The cause of heart failure was not identified during the first three admissions; however, on the fourth, transthoracic echocardiography (TTE) in the emergency department revealed loss of leaflet coaptation with severe MR, although the images were not recorded. Echocardiography performed shortly after admission demonstrated preserved leaflet coaptation, with MR reduced to no more than mild severity. At that time, clear evidence of pulmonary congestion prompted suspicion for eclipsed MR based on the clinical course. Norepinephrine stress echocardiography and other provocative tests were subsequently performed during hospitalization. Provocation testing was conducted under continuous blood pressure, heart rate, and echocardiographic monitoring of mitral leaflet coaptation and MR severity throughout stepwise norepinephrine infusion up to 0.1 μg/kg/min; however, severe MR with leaflet malcoaptation could not be reproduced. The patient remained on continuous cardiac telemetry monitoring throughout hospitalization, and no arrhythmic events coincided with the onset of the MR episodes. A Holter recording was not separately obtained. No further episodes occurred during hospitalization. Subsequent transesophageal echocardiography demonstrated moderate atrial functional MR (AFMR), which did not meet the surgical criteria, and the patient was discharged without intervention.

Ten days following discharge, while conversing with a visitor at home, she suddenly developed paroxysmal dyspnea, forcing her to adopt the recumbent position. Although symptoms improved partially, residual dyspnea prompted her to visit the emergency department the following day. On arrival, she was hemodynamically stable and could converse; nonetheless, after transfer to the echocardiography laboratory, TTE exhibited massive MR due to leaflet coaptation loss, accompanied by severe pulmonary hypertension and a drop in systolic blood pressure from 130 mmHg on arrival to 100 mmHg. Noninvasive positive pressure ventilation was initiated along with intravenous human atrial natriuretic peptide and furosemide. One hour later, repeat TTE demonstrated restored leaflet coaptation, with MR improving to a mild-to-moderate grade, and her symptoms promptly resolved.

Echocardiographic findings during the acute episode

**Figure 1:**
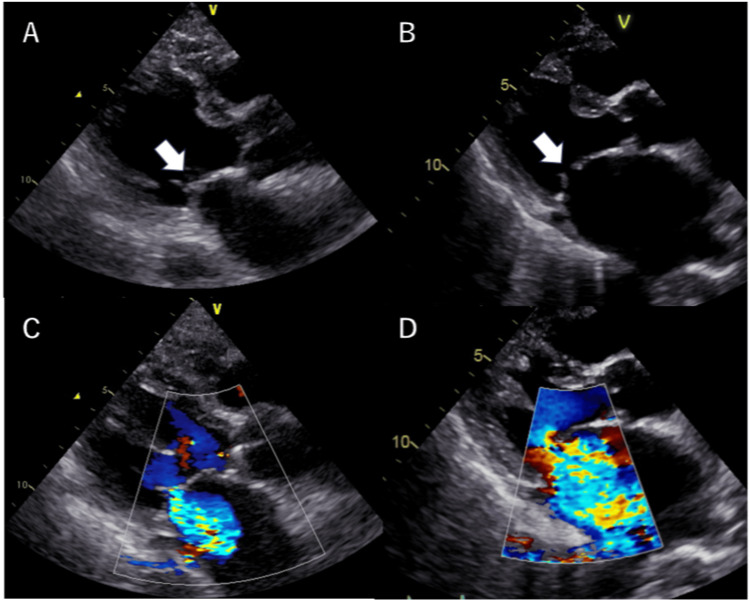
Parasternal long-axis transthoracic echocardiographic views comparing the period between episodes and the acute episode. A: Between episodes (color Doppler off), showing preserved mitral leaflet coaptation (arrows). B: During an acute episode (color Doppler off), demonstrating complete loss of coaptation between the anterior and posterior mitral leaflets (arrows). C: Between episodes (color Doppler on), showing only mild mitral regurgitation (MR). D: During an acute episode (color Doppler on), revealing severe MR with a dagger-shaped jet. MR, mitral regurgitation.

TTE performed during the acute episode revealed complete leaflet coaptation loss between the anterior and posterior mitral leaflets, resulting in severe MR with prominent systolic backflow (Figure 1).

**Figure 2:**
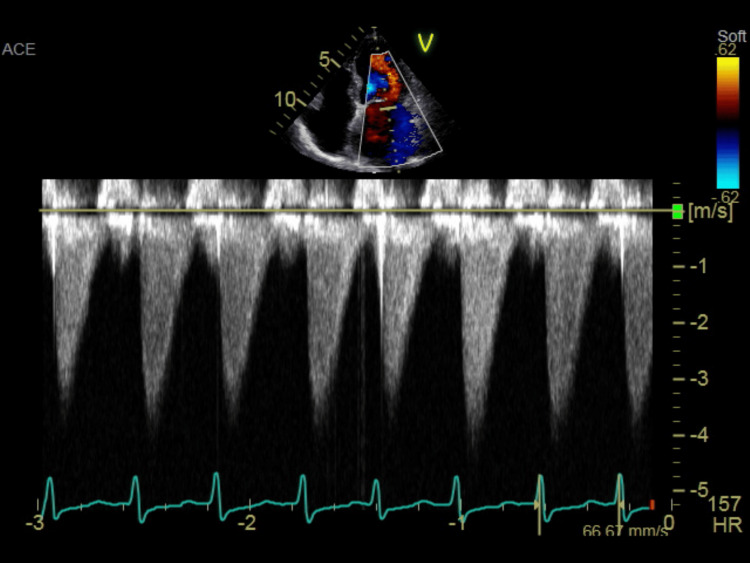
Continuous-wave Doppler during an acute episode. Continuous-wave Doppler of the MR jet demonstrates a characteristic dagger-shaped contour with an early-systolic sharp peak followed by rapid deceleration. MR, mitral regurgitation.

Continuous-wave Doppler of the MR jet demonstrated a dagger-shaped pattern with an early-systolic sharp peak followed by rapid deceleration (Figure 2).

Additionally, tricuspid regurgitation (TR) Doppler demonstrated a TR pressure gradient (TR-PG) of 57 mmHg during the acute episode, compared with 32 mmHg on presentation to the emergency department and 21 mmHg after symptomatic and hemodynamic improvement following treatment. These serial changes illustrate the abrupt and reversible increase in pulmonary artery pressure accompanying the MR episode. LVEF was preserved, and mitral annular dilation was not observed. Together, these findings suggest an acute and reversible mechanism of functional MR distinct from atrial functional MR, which is typically characterized by chronic progressive annular dilation.

Diagnosis and management

This patient had a history of multiple hospitalizations for acute decompensated heart failure with paroxysmal onset. Nevertheless, MR severity between episodes did not meet the surgical intervention criteria. On admission, TTE performed during an acute episode documented severe MR, establishing eclipsed MR as the heart failure cause. No significant structural abnormalities were noted in the mitral valve apparatus even during the episode, and LVEF was preserved. The results of blood cultures remained negative, inflammatory markers were not elevated, and echocardiography showed no vegetations, making infective endocarditis unlikely. Coronary angiography was performed and demonstrated intact coronary arteries, thereby excluding ischemic heart disease as a contributing factor. Despite optimal medical therapy, the patient experienced recurrent episodes, and no precipitating factors could be identified, prompting consideration of surgical intervention.

Although the patient was 82 years old, she was deemed operable based on her overall condition and cardiopulmonary reserve. Mitral valve replacement (MVR) was chosen over repair to achieve definitive recurrence prevention. While awaiting elective surgery during continued hospitalization, the patient developed another episode of massive MR at rest without any identifiable trigger, with complete leaflet coaptation loss. Consequently, early surgical intervention was deemed necessary, and urgent MVR was performed.

Operative findings

Following median sternotomy, cardiopulmonary bypass was established, and after cardioplegic arrest, the mitral valve was approached through a standard right-sided left atriotomy. The mitral valve exhibited only mild thickening of the anterior leaflet with no rupture, marked thickening, elongation, or shortening of the posterior leaflet, chordae tendineae, or papillary muscles. No significant annular dilation was observed. Intraoperative sizing indicated that a 27-mm prosthetic valve was the appropriate size, and leaflet mobility was well preserved. The anterior and posterior leaflets were excised and preserved, respectively, and MVR was performed using a 27-mm mosaic bioprosthetic valve (Medtronic, Minneapolis, MN, USA). The tricuspid valve showed mild annular dilation, and tricuspid annuloplasty was performed using a 28-mm contour 3D ring (Medtronic), which successfully controlled regurgitation.

Pathological findings

**Figure 3:**
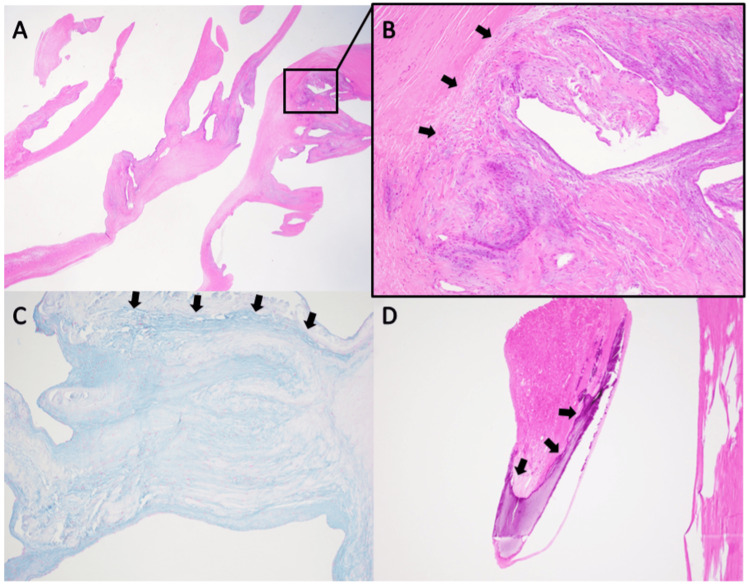
Histopathological findings of the mitral valve and papillary muscle. A: Low-power hematoxylin-eosin (H&E) showing preserved overall mitral leaflet architecture with only mild degenerative change. B: High-power H&E (magnified view of the boxed area in A) demonstrating myxomatous degeneration characterized by expansion of the spongiosa, myxoid matrix accumulation, and reduced elastic fibers (arrows). C: Alcian blue staining highlighting abundant acidic mucopolysaccharide deposition within the expanded spongiosa (arrows). D: H&E of the papillary muscle showing focal calcification (arrows). H&E, hematoxylin and eosin.

Histopathological examination of the excised mitral leaflet and papillary muscle, using hematoxylin-eosin and Alcian blue staining, revealed only mild fibrosis and myocyte hypertrophy in the subendocardial myocardium without myocyte disarray, inflammatory infiltrates, or amyloid deposition. The anterior mitral leaflet demonstrated Alcian-blue-positive myxoid degeneration as the predominant finding, with focal calcification and chordal fusion but no marked neovascularization or severe organic degenerative changes (Figure 3).

Postoperative course

The postoperative course was uneventful, without any perioperative complications or recurrence of MR or heart failure symptoms. Echocardiography before discharge confirmed good function of the prosthetic mitral valve and absence of MR and significant tricuspid regurgitation. The patient was discharged in a good condition on postoperative day 10. During follow-up, the patient remained free of recurrent acute MR and heart failure. Ten months post-surgery, the patient was readmitted for sepsis secondary to tuberculosis and subsequently died from the infection.

## Discussion

Eclipsed MR is characterized by a transient and severe functional MR that occurs abruptly and resolves spontaneously or with medical therapy, often without structural abnormalities in the mitral valve complex [1]. Our patient experienced multiple hospitalizations for acute heart failure; nevertheless, MR severity between episodes did not meet surgical criteria. Diagnosis was confirmed only when severe MR was captured on TTE during an acute episode. This rapid, complete resolution underscores the diagnostic difficulty and mirrors the experience of Milleron et al. [2].

From a mechanistic perspective, the lack of annular dilation or structural changes in the leaflets, chordae, or papillary muscles differentiates eclipsed MR from AFMR, which is characterized by chronic atrial enlargement, progressive annular dilation, and persistent MR [6]. Moreover, the dagger-shaped MR jet observed on continuous-wave Doppler and the abrupt rise in TR-PG during the episode reflect acute hemodynamic changes and pulmonary hypertension, findings not typical of AFMR. Eclipsed MR is also distinct from ventricular functional MR, which is primarily associated with ventricular remodeling [7]. These distinguishing features may help differentiate eclipsed MR from other forms of functional MR in clinical practice.

The potential contribution of systemic sclerosis should also be considered. Coronary microvascular dysfunction and myocardial fibrosis have been reported in systemic sclerosis and could theoretically impair papillary muscle function and alter mitral leaflet coaptation [11]. However, this patient had no evidence of active systemic sclerosis, and no findings directly supported such a mechanism. Therefore, any association between systemic sclerosis and eclipsed MR remains speculative.

The precise mechanism underlying eclipsed MR remains unclear. Previous reports have suggested multiple potential triggers, including abrupt changes in loading conditions, transient papillary muscle dysfunction, and fluctuations in left atrial pressure. In our case, continuous inpatient telemetry did not identify arrhythmic events coinciding with the onset of the MR episodes. In addition, provocation testing with norepinephrine infusion and volume loading failed to reproduce the episode, underscoring that a negative result does not exclude the diagnosis of eclipsed MR, an important clinical takeaway [4,5].

Furthermore, treatment selection for eclipsed MR is challenging [8]. Recurrent hospitalizations for acute pulmonary edema despite optimal medical therapy strongly indicated the need for definitive surgical intervention.

Although reports have described medical stabilization with vasodilators such as nitrates in rare cases [9], recurrence is common. Indeed, Oyama et al. reported a patient who experienced repeated episodes despite medical therapy and ultimately required mitral valve replacement [10]. Such cases highlight that surgical intervention is often necessary, as seen in our case. Therefore, the occurrence of severe MR episodes during hospitalization without identifiable triggers warrants early surgical management.

While mitral valve repair is generally preferred when feasible, operative inspection and pathology in our case confirmed only minimal structural changes, with no annular dilation or localized degenerative lesions. Thus, there was no clear target for durable repair, making a durable outcome with MVP unlikely. In addition, previous reports have documented recurrence after MVP in similar contexts [12], supporting the decision to proceed with replacement.

Although elderly, the patient's general condition and cardiopulmonary reserve were preserved; therefore, we prioritized long-term prevention of recurrence and selected MVR with concomitant tricuspid annuloplasty.

During follow-up after MVR, no recurrence of severe MR episodes or heart failure symptoms occurred, and MR remained well controlled. This suggests that appropriate surgical intervention may enable long-term symptom control in patients with eclipsed MR, and that in selected patients - particularly when repair durability is uncertain - valve replacement may be preferable to repair to ensure durable outcomes.

Nevertheless, several limitations should be acknowledged. As this is a single-case report, our findings cannot be generalized to all patients with eclipsed MR. Further accumulation of well-documented cases will be necessary to improve our understanding of its pathophysiology, refine diagnostic criteria, and guide optimal management.

## Conclusions

Eclipsed mitral regurgitation is a paroxysmal, reversible form of functional MR that is easily missed unless imaging coincides with symptoms; thus, echocardiographic documentation during an episode is pivotal, and a negative provocation test does not exclude the diagnosis. In our patient, TTE during an episode captured the transient loss of leaflet coaptation with a dagger-shaped MR jet, whereas MR was mild between episodes. Operative inspection and histopathology showed preserved leaflet architecture with only mild myxomatous change, indicating minimal intrinsic valvular disease. Because recurrent pulmonary edema occurred without identifiable triggers and there was no clear target for a durable repair, early surgery was undertaken; MVR with concomitant tricuspid annuloplasty provided durable control with no recurrence during follow-up. In selected patients - particularly when repair durability is uncertain - valve replacement may be preferable to repair. Accumulating well-documented cases will refine diagnostic criteria and management strategies.
